# Anogenital Distance and Phthalate Exposure: Swan et al. Respond

**Published:** 2006-01

**Authors:** Shanna H. Swan, Katharina Main, Robin Kruse, Sara Stewart, Bruce Redmon, Christine Ternand, Shannon Sullivan

**Affiliations:** University of Rochester, Rochester, New York, E-mail: shanna_swan@urmc.rochester.edu; University of Copenhagen, Copenhagen, Denmark; University of Missouri-Columbia, Columbia, Missouri; University of Minnesota Medical School, Minneapolis, Minnesota; University of Iowa, Iowa City, Iowa

In their letter, McEwen and Renner raise several points that we would like to discuss.

First, because all infants in our study (Swan et al. 2005) appeared normal, McEwen and Renner infer that there is no evidence of an adverse effect. However, the absence of evidence of an effect in infancy does not preclude serious adverse effects in later life. For example, the genital cancers that were identified in young women, on average 19 years after their prenatal exposure to the drug diethylstilbestrol, were seen in females who had appeared to be completely normal until that time (Herbst et al. 1971). In this case, unlike that example, we do have some evidence of anatomical changes in young boys. Although anogenital distance (AGD) has rarely been used as a measure of androgen action in humans, our data suggest that shortened AGD reflects reduced androgen action *in utero*; AGD was correlated with the degree of testicular descent and penile volume, and children with smaller AGD tended to have smaller scrotums; these are all signs of reduced androgen action.

McEwen and Renner state that the range of AGD reported in our study (Swan et al. 2005) is likely to be representative of normal study subjects. In fact, this information is not yet available because this is the first population-based study that utilized this measurement. AGD has, however, been used in the diagnosis of medical conditions such as congenital adrenal hyperplasia, in which AGD in females is increased by excess androgen exposure (Callegari et al. 1987). AGD is also known to be sexually dimorphic in humans as well as rodents (Salazar-Martinez et al. 2004).

McEwen and Renner point out that one previous study [*n* = 42; (Salazar-Martinez et al. 2004)] used an alternative measure of AGD in human infants. However, as we indicated in our article (Swan et al. 2005), this alternative definition is less precise than the one we used and does not correspond to the measure of anogenital distance most frequently used in toxicologic studies of rodents. Our use of this measure of AGD emphasizes the correspondence between traditional toxicology studies and our study.

In our study (Swan et al. 2005) we did not have data that would allow us to consider parental phenotype (e.g. parental height or father’s AGD), as McEwen and Renner suggest should be done. If AGD was affected by parental stature (through infant body size), this association should be controlled for by adjusting for body size. Moreover, in order for a phenotypic variable to explain the observed association, it too would have to be related to maternal phthalate levels. This, too, would be an interesting finding.

McEwen and Renner question the use of normalizing AGD by dividing by weight (AGI) at examination. We examined several alternative measures of body size and, as discussed in our article (Swan et al. 2005), AGI provided the best fit to the data (independent of phthalates). Vanderbergh and Huggett (1995) found the same to be true in rodents. The fact that there was some variation of AGI with age is to be expected; not all 1-year-olds have the same length, either.

McEwen and Renner point out potential sources of “exposure misclassification” which, we agree, may have been present (and we stated so) (Swan et al. 2005). However, unless these sources of measurement error were related to AGD, their presence would lead to underestimates of the strength of the associations we presented.

We examined a number of potential confounders, such as maternal smoking and alcohol consumption; the prevalence of both was quite low (Swan et al. 2005). None affected results appreciably. Of course, the phantom “unmeasured confounder” always lurks in the wings of any observational study, can never be ruled out, and is a favorite of critics of epidemiologic studies. Any constructive suggestions for alternatives to observational studies would be appreciated; the only alternative we know of, randomizing pregnant women to receive phthalates (or not), hardly seems ethical.

Rodent studies test only one phthalate at a time. As we demonstrated (Swan et al. 2005), women were exposed to measurable levels of multiple phthalates, many known to be reproductively toxic. Until we have data on the toxicology of this complex mixture, we do not have the information to draw conclusions about the relative toxicity of these compounds in rodents versus humans. Furthermore, although doses in rodent studies of specific phthalates are high, effects have been demonstrated at lower doses used in recent studies (Lehmann et al.). Unfortunately no toxicologic study has yet examined effects of phthalates at environmental levels. Because we did find a significant association with phthalates at such levels, we can only conclude that environmental levels, however low, are associated with somatic alterations in humans.

Our study (Swan et al. 2005) is relatively small and must be replicated; subsequent studies will undoubtedly eliminate many of the sources of potential exposure and outcome misclassification. Nonetheless, in this first study of its kind, we set out to test the hypothesis, suggested by a large toxicologic literature (Gray et al. 2000), that prenatal phthalate exposure is associated with several measures in humans that reflect the antiandrogenic action of these chemicals. Using similar outcome measures to those utilized in these toxicologic studies, that is what we found.
